# Characterizing the relation of functional and Early Folding Residues in protein structures using the example of aminoacyl-tRNA synthetases

**DOI:** 10.1371/journal.pone.0206369

**Published:** 2018-10-30

**Authors:** Sebastian Bittrich, Michael Schroeder, Dirk Labudde

**Affiliations:** 1 Applied Computer Sciences & Biosciences, University of Applied Sciences Mittweida, Mittweida, Saxony, Germany; 2 Biotechnology Center (BIOTEC), Technische Universität Dresden, Dresden, Saxony, Germany; Weizmann Institute of Science, ISRAEL

## Abstract

Proteins are chains of amino acids which adopt a three-dimensional structure and are then able to catalyze chemical reactions or propagate signals in organisms. Without external influence, many proteins fold into their native structure, and a small number of Early Folding Residues (EFR) have previously been shown to initiate the formation of secondary structure elements and guide their respective assembly. Using the two diverse superfamilies of aminoacyl-tRNA synthetases (aaRS), it is shown that the position of EFR is preserved over the course of evolution even when the corresponding sequence conservation is small. Folding initiation sites are positioned in the center of secondary structure elements, independent of aaRS class. In class I, the predicted position of EFR resembles an ancient structural packing motif present in many seemingly unrelated proteins. Furthermore, it is shown that EFR and functionally relevant residues in aaRS are almost entirely disjoint sets of residues. The Start2Fold database is used to investigate whether this separation of EFR and functional residues can be observed for other proteins. EFR are found to constitute crucial connectors of protein regions which are distant at sequence level. Especially, these residues exhibit a high number of non-covalent residue-residue contacts such as hydrogen bonds and hydrophobic interactions. This tendency also manifests as energetically stable local regions, as substantiated by a knowledge-based potential. Despite profound differences regarding how EFR and functional residues are embedded in protein structures, a strict separation of structurally and functionally relevant residues cannot be observed for a more general collection of proteins.

## Introduction

Most proteins adopt their three-dimensional conformation autonomously during the process of protein folding [1, 2]. Various diseases are caused by misfolding or aggregation of proteins [3–6]. During the protein folding process, the denatured chain of amino acids passes an energetic barrier, called transition state, to form a compact and functional structure [2].

How proteins fold is an open question [1]. There is a lack of experimental data describing which events or residues guide the folding process [7–9]. The protein sequence resembles the starting point and the three-dimensional structure captures the result of the protein folding process for a wide range of proteins, yet how they connect via transition states is unclear. The unstable nature of transition states hinders their experimental determination [10, 11]. Another obstacle for the understanding of the sequence-structure relation is that some proteins depend on chaperons to fold correctly [6].

### The defined-pathway model

Alternative folding pathways have been described for homologous proteins [12]. It is an open question if a general folding pattern can be derived which is relevant for all proteins [13]. Also, there is dispute which aspects of protein folding are stochastic and which are deterministic [14, 15]. The defined-pathway model proposes that small fragments fold first and then guide a step-wise assembly of further parts of the protein until the native structure is formed [14, 16, 17]. Such fragments fold autonomously—no other region of the protein directly supports or hinders their formation [14, 17]. Which parts of the protein initiate the formation of local, ordered structures, e.g. secondary structure elements, is encoded in their sequence [18–23]. Consequently, these regions decrease in free energy as well as entropy and stabilize the protein during the folding process [23, 24]. This also supports the observation that proteins fold cotranslationally as they are being synthesized by a ribosome and stabilizing tertiary contacts cannot be formed yet [25]. Tertiary contacts are formed between residues more than five sequence apart [26]. These local structures form tertiary contacts and assemble the global structure [14, 18, 22, 27, 28]. The formation of a native structure causes a further decrease in free energy [17, 29, 30]. Tertiary contacts are especially important for the stability of the hydrophobic core of the native structure [31].

### Identifying Early Folding Residues during protein folding

In recent years, various experimental strategies [32–35] were established which can identify residues crucial for the folding process. Pulse labeling hydrogen-deuterium exchange (HDX) [14, 31, 36–41] tracks the protein folding process with spatial and temporal resolution. The state of a protein can be controlled e.g. by denaturants or temperature [37]. Starting from a denatured protein, folding conditions are gradually established until the protein refolded completely. The resulting folding trajectory can be studied by HDX. Depending on the state of the folding process, individual amino acids will be susceptible to or protected from an exchange of the hydrogen atom of their amide group. Residues become protected when their amide group is isolated from the solvent as the effect of other residues surrounding them. When the folding process affects a residue, its spatial neighborhood is altered. Thereby, especially the formation of hydrogen bonds involving the amide group is relevant. Where and when these exchanges occur is tracked by a downstream mass spectroscopy or nuclear magnetic resonance spectroscopy. Residues which are protected from the exchange at the earliest stages [14, 39–41] are called Early Folding Residues (EFR). Residues which are protected only at later stages or not at all are referred to as Late Folding Residues (LFR). One can also argue that the experimental signal of EFR is currently too little understood. The protection of amide groups occur at an exceedingly fast timescale. In some cases, they may not be the effect of the formation of hydrogen bonds but rather be the mere result of undirected physical chemistry. Whether the conformations initially formed by EFR are present in the native structure is still unclear. Also, other experimental techniques for the determination of key residues in the folding process [32–35] show little correlation with the annotation of EFR [23]. E.g., data from *ϕ*-value analysis is difficult to interpret on its own [32] and may differ drastically depending on the introduced amino acid substitution, so no one-to-one relation between it and EFR can be expected [31] which pronounces the difficulties of studying the structural role of EFR. They were shown to initiate the folding process and the formation of secondary structure elements [41] or even larger autonomously folding units [14]. EFR tend to be conserved, but non-functional residues [42]. In contrast, LFR may be relevant during later stages of the folding process, implement protein function, or be mere spacers between protein regions. Experimentally determined EFR are provided by the Start2Fold database [41]. EFoldMine [9] is a classifier that predicts EFR from sequence. This allows the analysis of folding initiation sites in protein families for which no time-dependent HDX data exists.

### The evolutionary history of aminoacyl-tRNA synthetases

Aminoacyl-tRNA synthetases (aaRS) may be the proteins with the most intriguing evolutionary history and are a prime candidate to analyze as their emergence is well-discussed in literature [43–48]. aaRS enzymes attach amino acids to their cognate tRNA, which is subsequently recognized by its anti-codon and consumed by a ribosome. Thus, aaRS implement the genetic code and give insights into the earliest stages of life. For each amino acid, a dedicated aaRS implementation exists in each organism. E.g., AspRS attaches aspartic acid to tRNA^Asp^ in two-step reaction which involves the recognition of ATP, amino acid, and tRNA.

$$\begin{array}{c} \text{Asp}+\text{ATP}\overset{\text{AspRS}}{\to }\text{Asp}-\text{AMP}+{\text{PP}}_{\text{i}}\\ \text{Asp}-\text{AMP}+{\text{tRNA}}^{\text{Asp}}\overset{\text{AspRS}}{\to }\text{Asp}-{\text{tRNA}}^{\text{Asp}}+\text{AMP}. \end{array}$$

Specific aaRS implementations are referred to as type. The 20 types can be divided into two complementary classes which differ significantly at sequence and structure level, feature distinct reaction mechanisms, and occur in diverse oligomerization states. Some organisms may feature additional aaRS such as PylRS which makes pyrrolysine accessible to protein biosynthesis. In a recent study [48], two ligand binding motifs—the Backbone Brackets and the Arginine Tweezers—were identified as characteristic for each aaRS class. These motifs were furthermore linked to primordial implementations of both aaRS classes called protozymes [46, 47]. The Rodin-Ohno hypothesis [43] proposes that aaRS enzymes were once complementarily encoded by the same gene. This provides an elegant explanation for the emergence and peculiarities of contemporary aaRS classes [43, 46–48]. It is hypothesized that all aaRS genes originate from this primordial gene encompassing both protozymes. They diverged and improved in specificity, but their catalytic core has been conserved.

## Motivation

**Fig 1:**
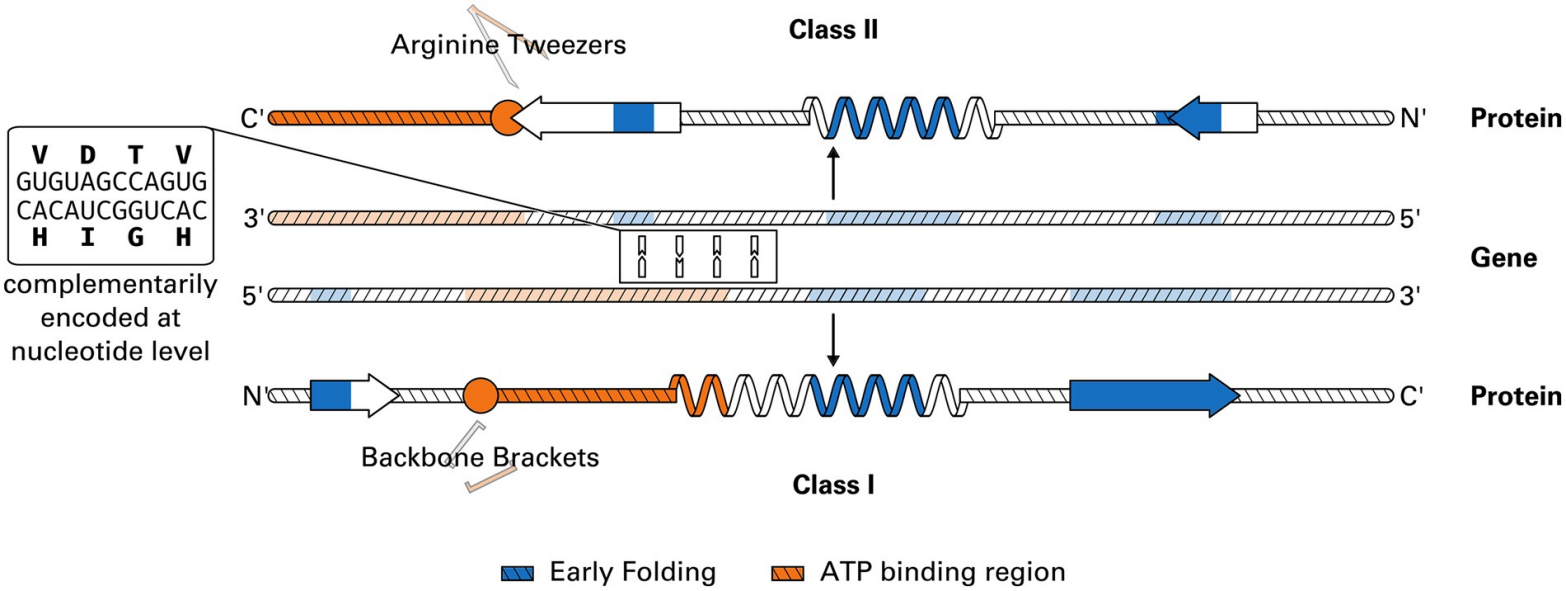
Schematic representation of protozyme regions. The two classes of contemporary aaRS enzymes may originate from opposite strands of the same gene. The corresponding peptides (called protozymes) have been shown to be catalytically active [43, 46]. The order of secondary structure elements in both protozymes resembles a mirror image. Using the EFoldMine classifier [9], EFR (i.e. folding initiation sites) were predicted (depicted in blue). EFR are a distinct set of residues with respect to ATP binding sites (orange) identified in a previous study [48]. Backbone Brackets and Arginine Tweezers are class-specific ATP binding motifs identified in the same study. Regardless of aaRS class, EFR occur in the center of secondary structure elements. Their position is preserved within aaRS classes despite sequence conservation being relatively small. The relative arrangement of EFR in class I resembles a prominent structural packing motif [50]. The more general Start2Fold dataset [41] is used to assess whether the separation of EFR and functional residues is a common theme in protein structures. The ATP binding region contains four binding residues each and was simplified to a continuous region for visual simplicity. Figure adapted from [46, 48].

It is unclear whether the position of EFR is consistent among homologues. Therefore, both superfamilies of aaRS were investigated and related to the two protozymes [43, 46] which may capture the primordial form of both classes. Interestingly, EFR are predicted [9] to occur in secondary structure elements (Fig 1) in a similar, yet mirrored fashion in both classes. Furthermore, functional ATP binding sites are located at distinct positions. EFR are likely structurally relevant for the correct protein fold [9]. This information is used to demonstrate that aaRS enzymes separate structurally relevant residues from functional residues. A modular design of proteins may improve evolvability because function can be changed without compromising the fold [49].

Several structural features are employed for a more general characterization of EFR using the Start2Fold database [41]. EFR exhibit lower, more stable computed energies in a coarse-grained energy model [29, 30]. Network analysis reveals that EFR are more connected to other residues and that they are located at crucial positions in the residue graph. This distinct wiring to the rest of the protein is especially furnished by hydrophobic interactions. Finally, it is shown that the separation of EFR and functional residues observed in aaRS is not present in all proteins. In particular, structures binding large ligands or other macromolecules are violating this characteristic.

## Results and discussion

**Fig 2:**
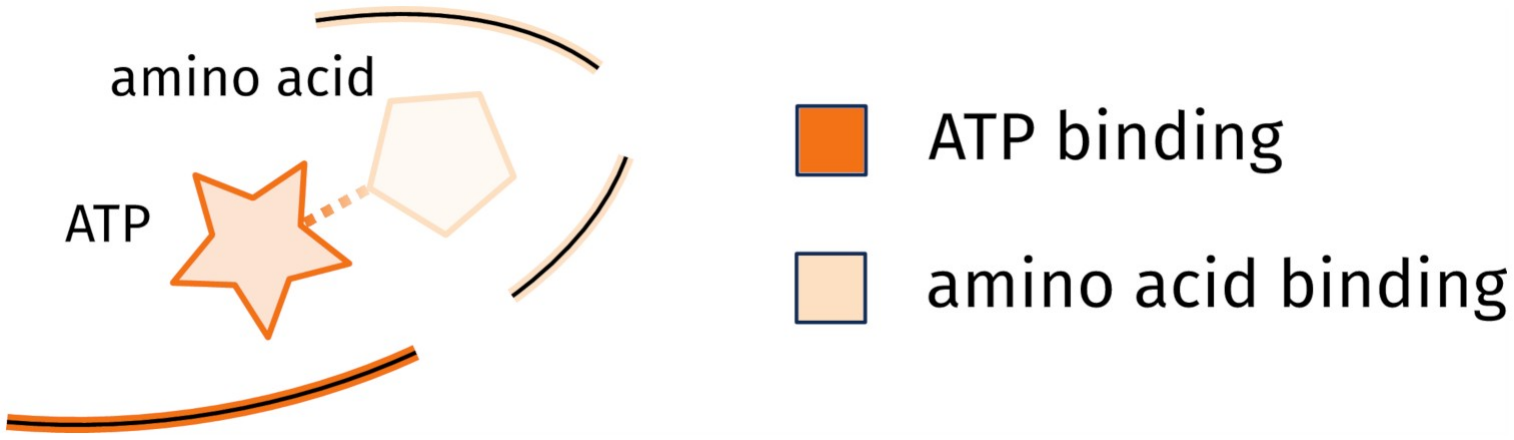
Binding site of aaRS enzymes. For the annotation of functional residues [48] the ligand binding site of aaRS structures was assessed. ATP binding (dark orange) is uniform within each aaRS class, whereas the amino acid binding (light orange) is specific to particular aaRS types such as AspRS. Figure adapted from [48].

Further analysis focuses on regions of today’s aaRS structures which correspond to the protozyme regions to assess how EFR predicted by EFoldMine [9] related to functional residues [48] in an evolutionary context. ATP and amino acid recognition sites were considered functional (Fig 2). Furthermore, we wanted to assess whether the predicted positions of EFR are consistent in these highly diverse superfamilies of enzymes. This analysis is backed by a manually curated dataset which accounts for high diversity of contemporary aaRS implementations [48].

### The position of Early Folding Residues is consistent in aminoacyl-tRNA synthetases

**Fig 3:**
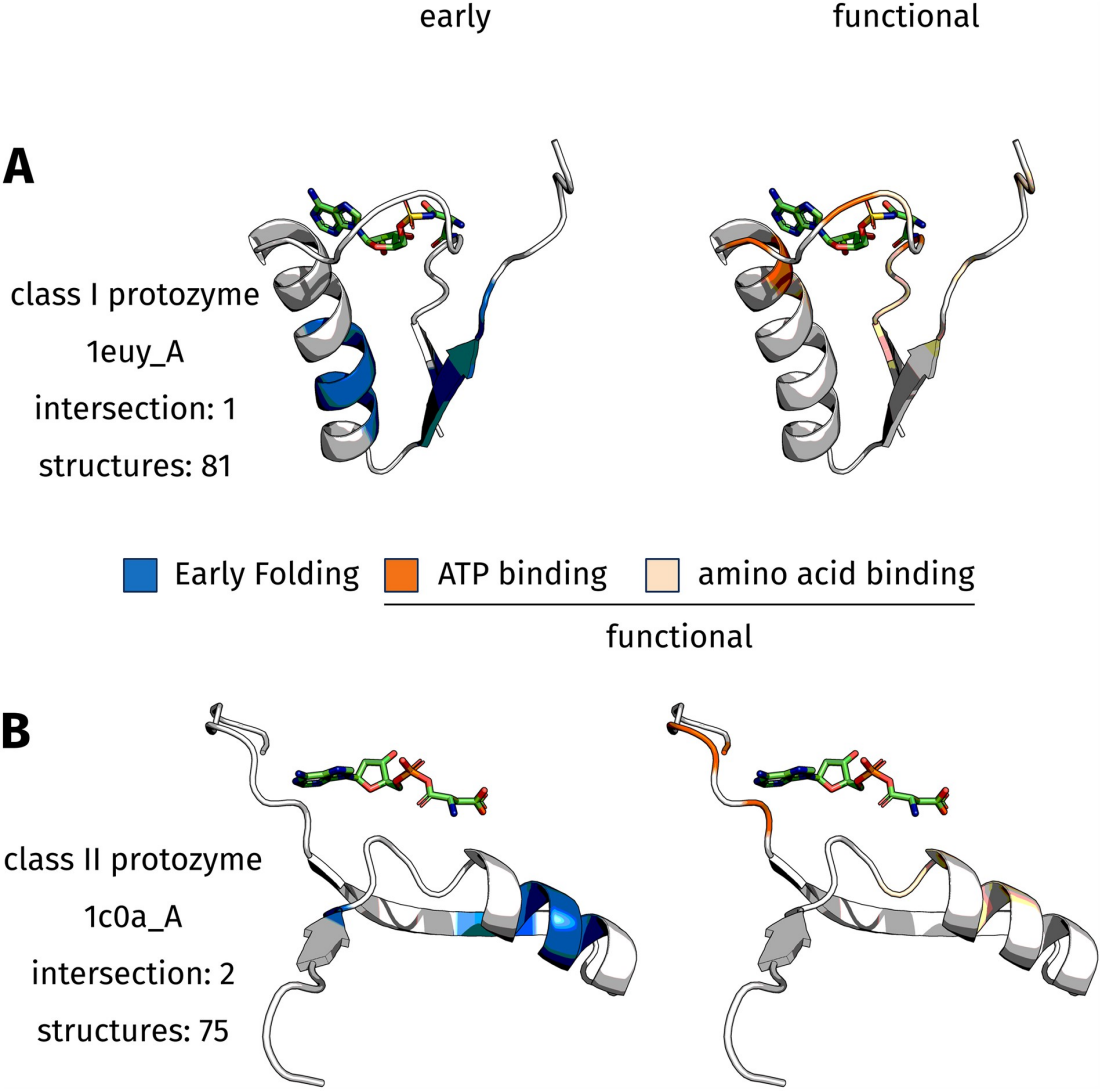
Protozyme regions of both aaRS classes. The protozyme regions [46–48] (in cartoon style) and the respective aminoacyl-AMP ligand (in sticks style) are depicted. This captures the state after the first reaction, when ATP and amino acid have been covalently bound. The ATP part is oriented to the left, whereas the amino acid is located on the right. Residues predicted to be Early Folding [9] are colored blue, whereas functional residues [48] are rendered in orange. ATP interaction sites are depicted in dark orange, residue positions observed to interact with the amino acid in any aaRS structure are rendered in light orange. In the rare cases that residues are both EFR and functional, they bind the amino acid part of the ligand in two specific aaRS types. (**A**) The class I protozyme is represented by truncated PDB:1euy_A. The respective EFR are located in the center of the ordered secondary structure elements and resemble a common structural packing motif that has been identified by Cammer & Carter [50]. In contrast, functional ligand binding sites are located in the upper part of each subfigure. They are primarily located in unordered coil regions. (**B**) The class II protozyme (represented by truncated PDB:1c0a_A) shows similar tendencies.

Fig 3 depicts the protozyme [46–48] of each aaRS class with an aminoacyl-AMP ligand present, which captures the intermediate of the enzymatic reaction. The shown secondary structure elements are extracted from the corresponding structures (PDB:1euy_A and PDB:1c0a_A), the protozymes have been shown to resemble molten globules [46, 47].

Analysis is based on 81 non-redundant structures for class I and 75 for class II, respectively. For each analyzed structure the corresponding sequence was used to predict the position of EFR [9]. A consistent numbering of residues within each class was established by a structure-guided multiple sequence alignment (MSA) [51]. Even within the depicted catalytic core of aaRS structures, sequences feature a high degree of variability and various inserts. Interestingly, residues predicted to be Early Folding are located at MSA columns which may not be extraordinarily conserved but are present in at least half of the corresponding sequences. EFR positions are mostly conserved among aaRS homologues. ATP binding sites are also consistent for the structures, whereas the position of amino acid binding sites varies. In the visualized protozyme regions (Fig 3), positions of EFR are located in ordered secondary structure elements. Functional residues, especially those realizing ATP recognition, are located in spatial proximity to one another. Furthermore, they occur in unordered coil regions and are located close to the ligand. ATP binding sites (dark orange) can be found on the left in proximity of the adenine part, whereas amino acid recognition sites (light orange) can be found on the right close to the amino acid part of the ligand. Sequence conservation scores were considered. For comparison, the highly conserved N-terminal arginine of the Arginine Tweezers motif exhibits a score of 11, scores close to 0 indicate no conservation. The average sequence conservation of the protozyme regions is 1.59 (1.42) for class I (and class II respectively). Positions predicted to be EFR exhibit scores of 2.50 (2.80). That for ATP binding sites is 3.75 (3.75) and for amino acid binding sites 1.85 (2.17). On average the EFoldMine prediction is 0.09 (0.09) for the protozyme regions. Positions considered EFR exhibit high values of 0.21 (0.20). ATP binding sites feature low scores, whereas amino acid binding sites feature slightly increased probabilities of being EFR (summarized in S1 Table). Detailed data for the annotated EFR and functional positions is provided in S1 and S2 Files. Because the position of amino acid binding sites is not consistent in the MSA, sequence conservation of these positions is relatively small. In contrast, ATP binding sites are mapped consistently in the MSA for both aaRS classes [48].

EFR exhibit smaller sequence conservation scores than ATP binding sites which indicates that more sequence variability can be tolerated for folding initiation sites. Protein function depends on particular amino acid side chains [52], whereas protein structure and secondary structure element formation is mainly the consequence of the hydrophobicity of amino acids [53, 54]. ATP binding sites exhibit lower EFR prediction scores compared to the average in the protozyme region which captures their tendency to occur in exposed, unordered coil regions as observed in the previously reported findings [48].

### Structural packing motif in class I aminoacyl-tRNA synthetases

The LFR position 284 features a remarkably high sequence conservation of 10. This position is part of the HIGH sequence motif which relates to ATP binding and the stabilization of the transition state [44]. In most class I aaRS, the HIGH motif is located at the N-terminal end of an *α*-helix. This particular arrangement is commonly observed for ATP binding proteins due to the favorable interactions between the negative charge of phosphate moieties and the dipole moment of the helix backbone [55]. Despite the defined secondary structure in this region, the HIGH motif is predicted to consist of LFR. EFR are located close to the C-terminal end of the helix (Fig 3A). Such folding initiation sites will lead to an extension of the nascent secondary structure element until certain sequence compositions terminate the process [7, 8]. Within this secondary structure element crucial for function, residues initiating its formation and residues binding the ligand occur at distinct positions. Furthermore, the observed C-terminal aggregation of EFR and the proximity to other EFR in neighboring *β*-strands substantiates a previously described structural packing motif in the catalytic core of class I aaRS. It is one of the most ancient and most widely distributed structural motif and was identified in a diverse set of proteins which encompasses the catalytic domain of class I, the anti-codon-binding domain in class II, and five other members of the Rossmanoid family [50]. This motif has been associated to a structural rearrangement important for function [56, 57]. The nearby Backbone Brackets motif rearranges upon ligand binding which implies that the structural rearrangement observed is a feature common to all class I aaRS structures [48].

### Early Folding Residues are non-functional in aminoacyl-tRNA synthetases

In class I (visualized by truncated PDB:1euy_A), position 311 is the only residue which is both EFR and functional (Table 1). This position is only functional in TrpRS and TyrRS where it realizes binding of the respective amino acid. Both tryptophan and tyrosine are large, aromatic amino acids and it is hypothesized that they were added to the genetic code recently [45]. This implies that these EFR became functional late during the evolution of aaRS. The clear separation with respect to ATP recognition implies that the unifying aspect of all aaRS is binding of the ATP ligand and catalysis at the respective *α*-phosphate [48]. At first protozymes where required to bind ATP and later the amino acid binding sites improved in specificity, allowing them to discriminate between amino acids more reliably. Position 274 corresponds to the N-terminal residue of the Backbone Brackets structure motif. Close to this position various amino acid binding sites can be observed in other class I aaRS, while EFR are further away (S1 File). Despite being functionally relevant, the sequence conservation of position 274 amounts to 3 and is relatively small. This residue has been shown to realize ATP binding by backbone hydrogen bonds which can be virtually realized by all amino acids [48]. Thus, change can be compensated at this position as along as the backbone atoms can still bind the ATP ligand. Furthermore, this position interacts with the *α*-phosphate position of the ligand to which the aaRS attaches the proper amino acid [48]. Therefore, it is intuitive that many positions involved in amino acid recognition are located at neighbored sequence positions. In class I, 15 of 16 EFR positions in the MSA relate to well-mapped positions (i.e. present in >50% of aligned sequences).

**Table 1:** Comparison of folding characteristics and functional relevance for aaRS classes.

class	early	ATP	aa	ATP int.	aa int.	ATP shift	aa shift
class I	16	4	13	0	1	-0.95%	-1.87%
class II	10	4	8	0	2	-0.82%	1.22%
	26	8	21	0	3	-0.90%	-0.39%

ATP refers to the number of ATP binding sites and aa refers to the number of positions realizing amino acid recognition in any aaRS implementation. The intersection of functional residues involved in ATP and amino acid binding is given. The shift in probability to the expected intersection is stated (see Methods). Negative values occur when the observed intersection is smaller than that expected by the individual frequencies of EFR and functional residues. Positive values are observed when the overlap is more pronounced than to be expected. EFR and ATP binding residues are strictly separated, no residues share both labels. Also, positions relevant for amino acid specificity are remarkably well separated from EFR most of the time. The overlap is present in the amino acid recognition sites in two implementations respectively: TrpRS and TyrRS in class I as well as AspRS and PylRS in class II.

In class II, positions 665 and 666 are both functional and predicted to be EFR (Table 1). Again, these positions are not functional in most class II aaRS. Only in AspRS and PylRS they are observed to bind the amino acid part of the ligand. In agreement with the observation for aaRS class I, asparagine and pyrrolysine are relatively large ligands which may require EFR to participate in protein function. 9 of 10 EFR positions are well-mapped in class II. For both classes, functional positions are well-mapped too. For position 698 of class II a sequence conservation score of 11 is observed. This position is the N-terminal residue of the Arginine Tweezers motif [48] which has been demonstrated to depend on the conservation of this amino acid for ATP binding via salt bridges and *π*-cation interactions. Like in class I, ATP binding positions can be found accumulated together at sequence level without any EFR between them (S2 File). In summary, the position of folding initiation site is preserved in aaRS despite their large evolutionary divergence. Potentially, aaRS even had influence on the organization of the genetic code and may caused a shift in the interpretation of genetic information. Amino acids handled by class I more often constitute the hydrophobic core of proteins, whereas amino acids handled by class II are more likely to occur at the interface to the polar solvent [58].

### Early Folding Residues are hubs in protein structures

The Start2Fold dataset [41] is utilized to analyze EFR in more detail. It is investigated whether the observed separation of EFR and functional residues is specific for aaRS or can be observed for a diverse set of proteins. The Start2Fold database provides an annotation of EFR derived from experimental data. 2,966 residues in 27 proteins were analyzed. Used identifiers of the database are provided in JSON format in S3 File. General information is provided in S4 File. In particular, a coarse-grained energy model [29, 30], network analysis, and non-covalent contacts [59] are considered (see Methods). Correlations between used residue descriptors are provided in S1 Fig. EFR are biased to be in the core of the protein [41]. Thus, it was assessed if change for a feature is significant for the subset of buried residues according to their relative accessible surface area (RASA) [60].

**Fig 4:**
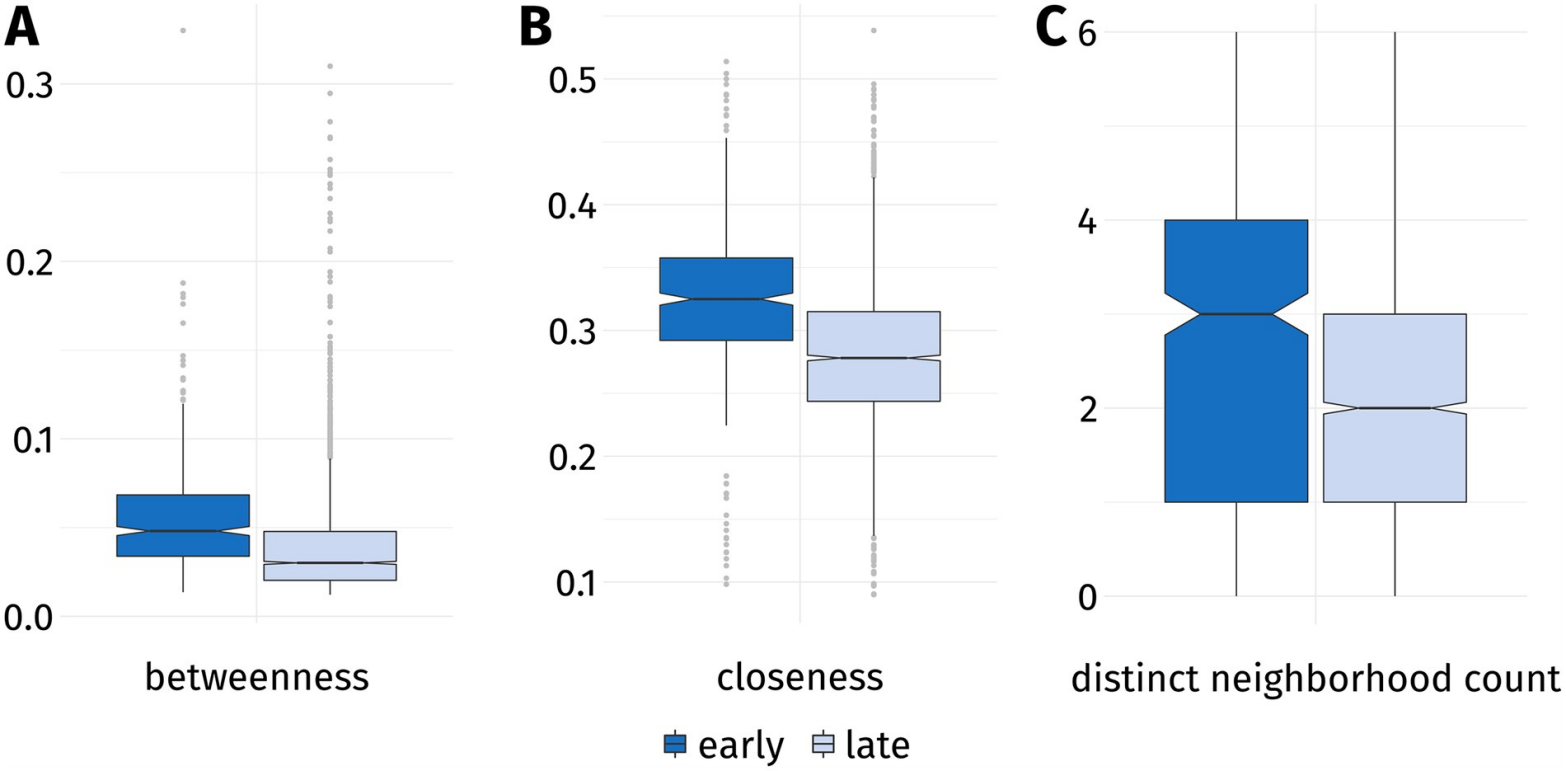
Topological properties of EFR and LFR. Proteins were represented as residue graphs and a network analysis was performed. The notch of a box corresponds to the confidence interval around its median: two notches which do not overlap indicate a difference of medians. (**A**) EFR have higher betweenness values implying that shortest paths in the graph tend to pass through these nodes more often. (**B**) They also exhibit higher closeness values because their average path length to other nodes is lower on average. (**C**) The distinct neighborhood count of a residue describes to how many separated regions it is connected. Residues are considered separated when their separation at sequence level is greater than five. EFR connect significantly more distant regions of a protein than LFR.

Graph representations of proteins are commonly employed to describe aspects of protein folding [11, 61]. Hydrophilic and aromatic amino acids have been found to be crucial connectors in the graph—so-called hubs—which underlines their importance in the context of protein folding [62]. Regarding the topological properties of residues derived from network analysis (see Methods and S2 Fig for a graphical depiction), EFR show a higher interconnectedness than LFR. They exhibit higher betweenness (Fig 4A) and closeness (Fig 4B) values. High betweenness values are observed for well-connected nodes which are passed by many of the shortest paths in a graph. High closeness values occur for nodes which can be reached by relatively short paths from arbitrary nodes. The distinct neighborhood count expresses to how many sequentially separated regions of a protein a residue is connected. Again a significant increase can be observed for EFR (Fig 4C). The clustering coefficient of a node is the number of edges between its adjacent nodes divided by the theoretical maximum of edges these nodes could form. The difference is insignificant in that case (see S2 Table). Regarding non-covalent contacts [59], EFR on average participate in 3.85 hydrogen bonds and form 1.31 hydrophobic interactions with other residues. This constitutes a significant increase compared to LFR. Energy values predicted from sequence using the eGOR method [29] are lower for LFR which indicates that the position of EFR is the consequence of the sequence composition of small fragments. The computed energy values of EFR are also significantly lower than the values of LFR which points to them participating in several favorable interactions.

The betweenness property is closely related to the small-world characteristics of networks (i.e. they are well-connected even when between most nodes no edge exists) and captures the specific ratio of protein surface and volume [61]. Residues relevant for the folding process have been shown to exhibit high betweenness values in the transition state and are crucial for the formation of the folding nucleus [61]. Interestingly, the clustering coefficient shows no difference between EFR and LFR when only buried residues are considered. Also, the value is higher for LFR, which is probably an effect of EFR being hubs which connect several separated regions of a protein (as shown by the distinct neighborhood count). These regions themselves are not well-connected, which results in a lower clustering coefficient for EFR. Especially tertiary contacts are also observed with an increased frequency for EFR, which also connects to the increased interconnectedness of EFR regarding computed energy values and non-covalent contacts. Secondary structure elements such as helices interact e.g. by hydrophobic interactions [63] and these tertiary contacts can unveil convergence or divergence in protein structures otherwise difficult to spot [50].

The performed network analysis aids the understanding on the idiosyncratic properties of EFR in the context of the whole protein and is in agreement with previous studies [10, 61, 62]. EFR being hubs between sequentially distant protein regions underlines their importance for the correct assembly of the tertiary structure. Orthogonal techniques not based on graph theory may be used to identify hubs in protein structures [50, 64]. Also, the set of considered residue descriptors can be enhanced: e.g. the RASA value has been shown to be temperature dependent and improved metrics have been published [58, 65]. Nevertheless, the increased number of local and tertiary contacts of EFR point to their importance for the whole protein folding process as described by the defined-pathway model [14, 17].

### Early Folding and functional residues exhibit distinct features

Division of labor is one of the most successful strategies observed in biology [42, 46, 47, 49, 66–68]. The separation of residues crucial for folding and those furnishing function may allow reuse of established protein folds [34, 42, 49, 68–70]. The sequence and structure space ascertained over the course of evolution seems small for a truly random exploration. Reusing established folds could also avoid slow-folding sequences or those prone to aggregation [33, 69, 71, 72]. A separation of folding initiation sites and functional residues may increase the evolvability of proteins [49, 70, 73]. Functional residues [52] can be mutated and new functions can be adopted without compromising the fold of the protein [34]. In consequence, a clear division should be observable between EFR—which initiate and guide the folding process—and the functional ones implementing protein function.

**Fig 5:**
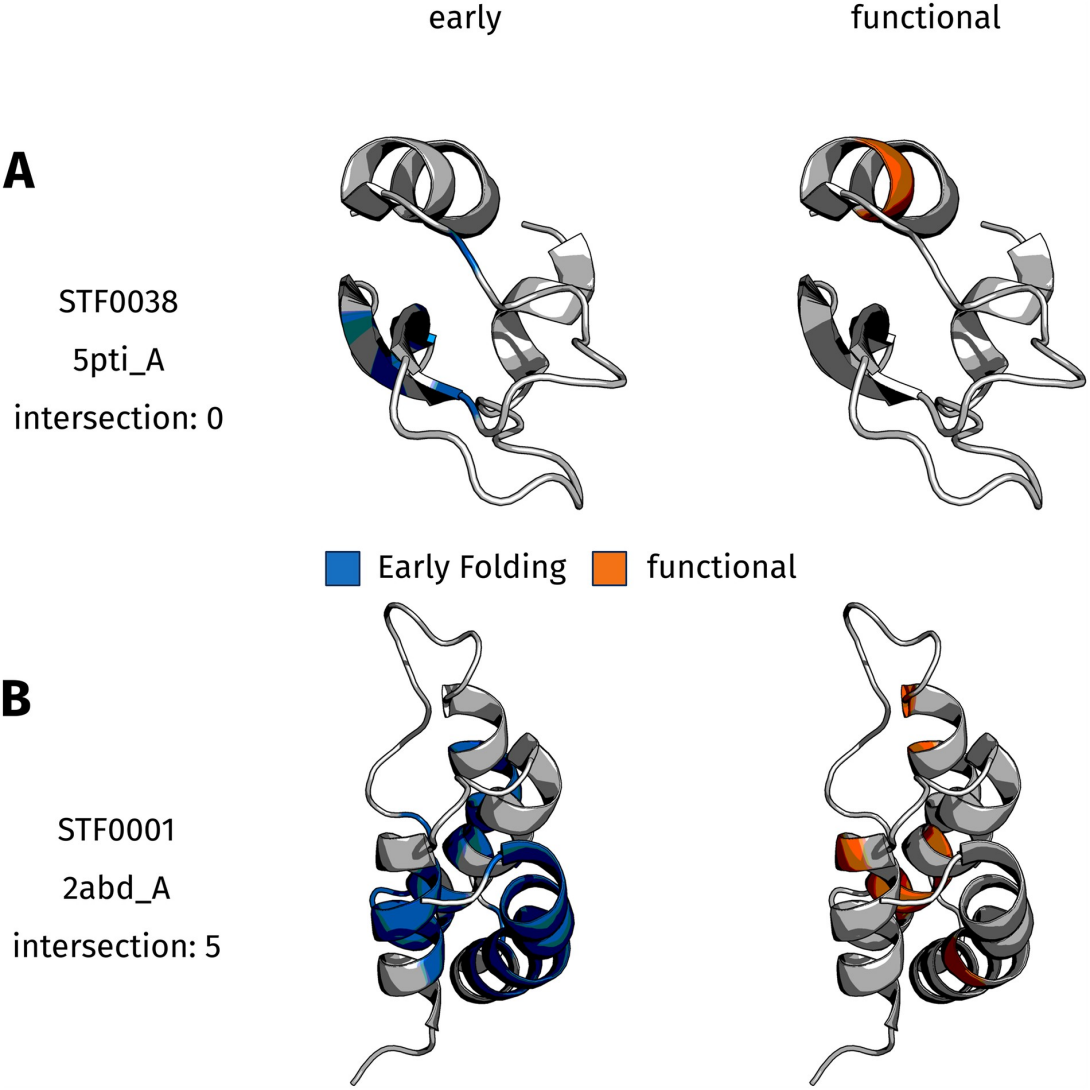
Rendered structures of 2 dataset entries. EFR are rendered in blue, functional residues are rendered in orange. (**A**) In the case of trypsin inhibitor (PDB:5pti_A) the intersection of EFR and functional residues is empty. For many proteins in the dataset, there is a clear distinction between both classes and structurally relevant residues have a propensity to be located in the core, while functional residues are exposed on the surface of a protein. (**B**) Five residues are both EFR and functional in the acyl-coenzyme A binding protein (PDB:2abd_A) which is one of the cases in the dataset where many residues are both EFR as well as functional.

To address this question, residues in the Start2Fold dataset [41] were labeled as either EFR or LFR as well as being either functional or non-functional. Active sites and ligand binding regions are considered to be the functional parts of proteins. The distribution of both binary variables (Table 2) shows that the majority of residues in the dataset are neither EFR (86.1%) nor functional (93.9%) residues. 0.9% share both classes, whereas 0.8% are expected to share both classes if their association was random (see Methods for details). The distribution of both variables separated by individual proteins is presented in S3 Table. For many proteins, no residues are both EFR and functional (Fig 5A). Furthermore, EFR tend to be located in the core of proteins, whereas functional residues are exposed towards the solvent in order to realize their respective function (Fig 5). The acyl-coenzyme A binding protein (STF0001) [35, 74, 75] features five residues which are both EFR and functional (Fig 5B). Another case where the overlap is large is T-cell surface antigen CD2 (STF0009) which can bind other protein antigens.

**Table 2:** Contingency table of folding characteristics and functional relevance.

	functional	non-functional
early	22	324
late	130	2,014

Out of 2,490 observations, 0.9% are EFR and functional at the same time. Based on the presented frequencies, 0.8% of all residues are expected to share both labels if their association is independent. This captures that a separation of EFR and functional cannot be observed in general. Proteins were excluded when no annotation of functional residues existed.

The acyl-coenzyme A binding protein may exhibit five residues which are both EFR and functional because it is a rather small protein of 86 residues which binds ligands with large aliphatic regions. Intuitively, the residues furnishing the bowl-like shape of the protein are also those which participate in the function of ligand binding [35, 74, 75]. Roughly half the residues of the acyl-coenzyme A binding protein are marked as EFR which further accentuates why the separation is less strict in this case. The primary selection pressure during evolution is on protein function [76] rather than on structural integrity [77]. In cases where a certain position is crucial for function, slower folding is tolerated which implies that structure and folding are subordinated to function [73]. Disordered proteins are another example of proteins without structural integrity which achieve a high robustness of function [78]. In structural biology, structure is considered to be a scaffold which allows proteins to implement a particular function [76, 78]. During evolution, it is most important that proteins retain their function [76, 79] and this may even require an explicit lack of a defined structure or structural flexibility [80].

**Fig 6:**
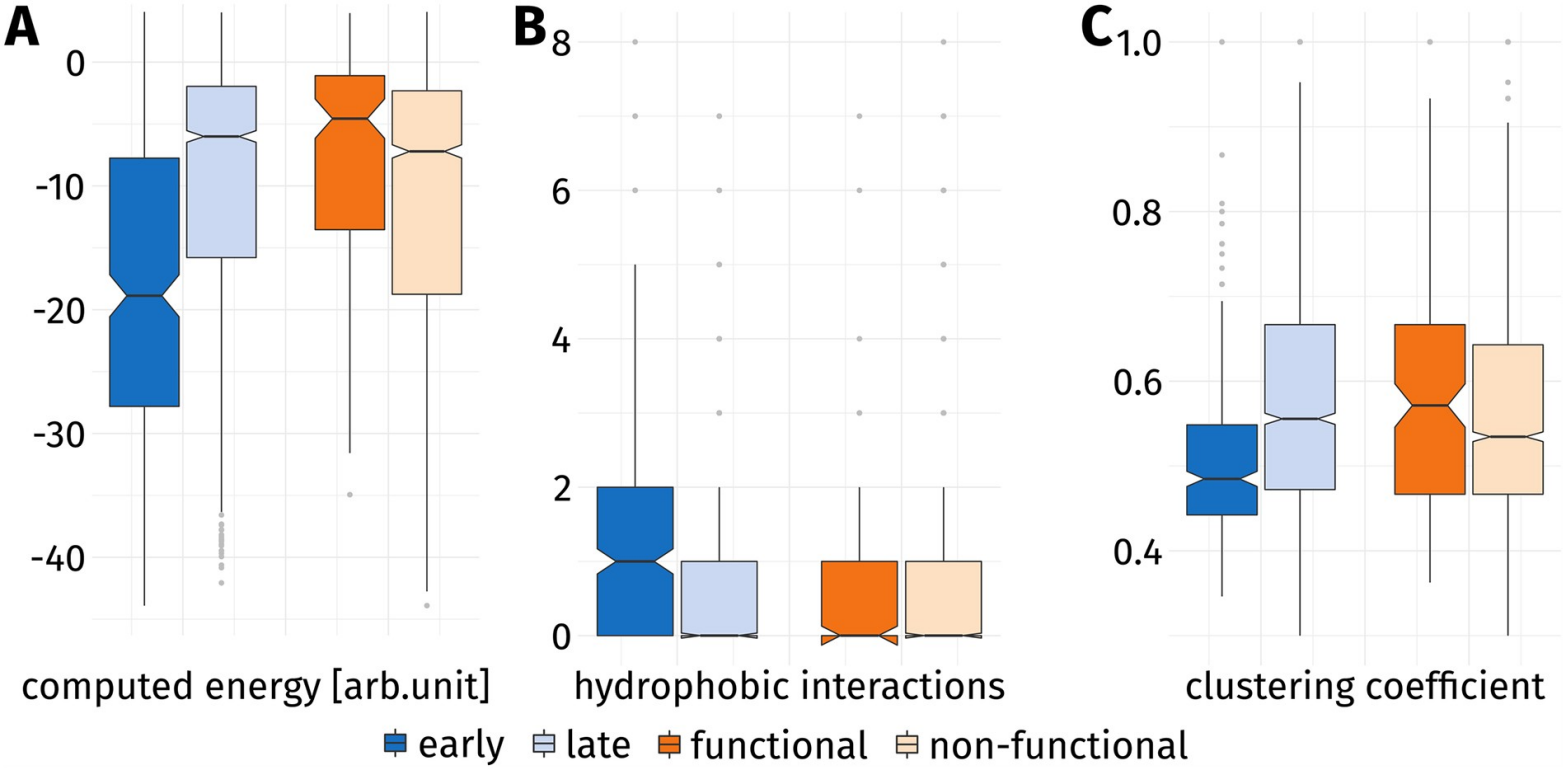
Characteristics of EFR and functional residues. EFR (dark blue) and LFR (light blue) are compared to functional (dark orange) and non-functional (light orange) residues. The notch of a box corresponds to the confidence interval around its median: two notches which do not overlap indicate a difference of medians. (**A**) EFR show lower computed energies than they are in contact with many residues and tend to be embedded in the hydrophobic core. In contrast, functional residues are exposed to the solvent in order to constitute e.g. binding sites. (**B**) Hydrophobic interactions occur especially in the core of a protein, thus, most residues do not form any. However, EFR show a significant increase compared to LFR. (**C**) The clustering coefficient of a node describes how well-connected its adjacent nodes are. EFR connect regions of a protein which are separated at sequence level and, thus, are not well-connected on their own. Functional residues exhibit higher clustering coefficient indicating a more connected set of adjacent nodes.

Several features were employed to substantiate the potential separation of structure and function at residue level (S4 Table). EFR show significantly lower computed energies when compared to LFR or functional residues (Fig 6A). Functional residues exhibit higher computed energies than their non-functional counterparts. Most residues form only a small number of hydrophobic interactions, however, the number for EFR is significantly increased (Fig 6B). 97.6% of EFR form hydrogen bonds and 65.1% participate in hydrophobic interactions. Functional residues participate to 88.8% in hydrogen bonds and to 39.5% in hydrophobic interactions. On the contrary, the change between the hydrogen bond count of EFR and functional residues in a buried state is insignificant. The clustering coefficient of a node captures how many edges can be observed between the adjacent nodes and, thus, describes how well-connected the direct surroundings of a node are. Functional residues show an insignificant change regarding this property. In contrast, the clustering coefficient significantly decreases when EFR are compared to LFR or functional residues (Fig 6C). In summary, EFR exhibit distinct properties compared to functional residues. Their surrounding secondary structure elements, values in Energy Profiles, and the number of hydrophobic interactions is especially characteristic. In terms of evolutionary information, functional residues exhibit a significant change compared to non-functional residues. Evolutionary information of functional residues amounts to 43.39 compared to 42.40 for EFR. LFR and non-functional residues are less conserved at sequence level.

Due to their purpose, EFR are located in the hydrophobic core and functional residues are primarily exposed to the solvent. These distinct requirements manifest in the computed energies. Furthermore, protein function can commonly be broken down to amino acids which feature hydrophilic, chemically functional groups [52]. Hydroxyl groups are prominent examples for functional groups contributing to catalysis [52]. Thus, functional residues are likely to exhibit above average computed energies because of their higher propensity to contain hydrophilic side chains. EFR tend to be crucial connectors in proteins, however, their clustering coefficient is low. This can be attributed to the fact that EFR connect many distinct neighborhoods. It was shown that functional residues have special requirements on how they are wired to the rest of a protein [81]: surrounding residues ensure the correct placement of functional residues [64, 81, 82], modulate their chemical properties such as the acid dissociation constant [52, 81, 83], or propagate signals to other parts of a protein [81]. Analogously, the evolutionary pressure on functional residues is increased compared to EFR and non-functional residues as indicated by the evolutionary information (S4 Table). In particular, catalytic activity of amino acids can be broken down to functional groups of their side chain [52]. The hydroxyl side chain of serine may be substituted by threonine or tyrosine. In contrast, contacts which stabilize protein structures can be primarily broken down to the hydrophobic or hydrophilic character of amino acids [53, 54] which allows for a wider range of tolerated mutations. Early stages of protein folding sample transient conformations [14, 23] and settle for stable, local structures. It has been shown that the characteristic of EFR is not directly linked to individual amino acids but rather the effect of the sequence composition of sequence fragments [9, 23, 40]. This may be another explanation why EFR are less conserved at sequence level than functional residues. That the folding nucleus of proteins is not necessarily sequentially conserved has been demonstrated previously [14, 84, 85], and makes it even more remarkable that sequence co-variation techniques such as the direct coupling analysis perform so well for structure prediction tasks [86, 87].

Modularity in proteins is also present in domains [68], secondary structure elements, and autonomous folding units of the defined-pathway model [17, 28]. Particularized knowledge of EFR may improve synthetic biology and could allow the design of proteins combining existing functional domains without influencing one another negatively [2, 49, 68, 88]. Furthermore, understanding the differences of structurally relevant residues and those implementing function could help in predicting mutation effects and provide a new level of detail by allowing whether a mutation disrupts the fold or the function of a protein [89, 90].

## Conclusion

A dataset [48] of aminoacyl-tRNA synthetase structures is analyzed. It is shown that the position of folding initiation sites is preserved over the course of evolution even when the corresponding sequence conservation is small. Folding initiation sites occur in the center of secondary structure elements, independent of class. Furthermore, the findings related to the protozymes of aminoacyl-tRNA synthetases substantiate that protein function can be considered the most important aspect of a protein [76] and retaining protein fold may be of subordinate importance [77]. Functional residues (i.e. ATP binding sites consistently shared by all types) exhibit a higher sequence conservation than Early Folding Residues. Early Folding Residues and functional residues are distinct sets of residues when amino acid binding positions are ignored which are only relevant in a small number of implementations. Even when these amino acid binding positions are considered to be functional in all implementations, the intersection is remarkably small for class I. In both superfamilies, Early Folding Residues are located consistently in the same columns of the respective MSA which agrees with the observation that this characteristic depends on the composition of local sequence fragments [9] and is relatively insensitive to inserts.

In the dataset of aminoacyl-tRNA synthetases, folding initiation sites and functional residues are separated. However, this seems not to be a universal characteristic of proteins as demonstrated on the more general Start2Fold dataset [41]. Nevertheless, understanding the topological differences of Early Folding Residues and functional residues provides insights into the way certain residues interact with the rest of the protein to fulfill their respective role. Early Folding Residues are an excellent tool to gain insights into the folding process with spatial and temporal resolution. Future studies may link them to characteristics at sequence level to understand which sequence composition causes particular regions of a protein to initiate the folding process. Features presented in this study are shown to be discriminative for Early Folding Residues. Classifiers for them based on sequence [9] or structure may annotate residues crucial for protein folding. Trained classifiers can also report as well as visualize the most discriminative features [91, 92] which may further delineate Early Folding Residues. This information is also invaluable for mutation studies, *ϕ*-value analysis, or protein design and can serve as basis for the prediction of mutation effects [89].

For decades, scientists longed for a glimpse into the folding process [7–9] and the analyzed datasets provide just that. The experimental signals [41] of early folding events are still difficult to interpret and the analyzed dataset may not be generalizable for large proteins, but the observations indicate that Early Folding Residues not only initiate the folding process, but are also relevant for the stabilization of the native structure. By relating this experimental data to evolutionary related proteins such as aminoacyl-tRNA synthetases, it is shown how folding initiation sites are conserved.

## Methods

### Creation of the aminoacyl-tRNA synthetase dataset

An evolutionary trajectory of highly diverse proteins can be found in aaRS. A detailed description of the methodology can be found in a previous study [48]. 972 aaRS structures from the PDB were analyzed. All structures were renumbered within each class which allows to directly compare structures: e.g. the first residue of the Backbone Brackets motif is at renumbered position 274 and can by found by that residue number in all other class I structures despite the high sequence variability. From these renumbered protein chains, the corresponding sequence was extracted and used as input for the EFoldMine algorithm [9] which predicts the probability of residues being EFR. This was necessary because no experimentally derived folding characteristics are available for aaRS proteins. Predicted scores exceeding 0.163 where considered EFR; this value has been shown to optimally separate EFR and LFR [9]. The annotation of functional residues was derived from a curated annotation of ligand binding sites [48]. For ligand binding, ATP binding sites and amino acid binding sites were distinguished as detected by PLIP [59]. Protozyme regions were extracted from PDB:1euy_A and PDB:1c0a_A to represent aaRS class I and II. This selection was for visualization purposes only and focused on structures with aminoacyl-AMP ligands. Selected residue numbers of the protozymes are 255–336 and 648–718, respectively [48]. The sequence conservation in aaRS sequences was computed by Jalview [93, 94] using only sequences which were used as input of the MSA. Positions composed of sets of amino acids with similar characteristics result in high values. The referenced MSA [48] was also used to pair both protozyme regions complementarily to create the schematic representation in Fig 1. Furthermore, the observed intersection between EFR and functional residues was expressed as probability and compared to the expected probability of a residue to share both the EFR and functional label based on their respective probabilities to occur individually.

### Creation of the Start2Fold dataset

Folding characteristics of residues were obtained from the Start2Fold database [41]. Therein, the authors adopted the definition of EFR from Li et al. [31] and presented a refined dataset which ignores possible back-unfolding and aggregation events [95]. The database covers all structural protein families present in CATH and SCOP [9]. However, the size of the deposited proteins [9, 23] varies from 56 to 164 residues (S3 Table) which makes this resource primarily relevant for the folding of similarly small proteins. Because local sequence features determine where EFR are located, this characteristic may be independent of sequence length and applicable for a wider range of proteins. The original dataset contains two groups of similar sequences of lysozymes (PDB:1hel_A, PDB:1lz1_A, PDB:2eql_A) and apo-myoglobins (PDB:1mbc_A, PDB:1ymb_A). In these cases, we only considered the structure with the highest resolution. This procedure resulted in a dataset for EFR characteristics encompassing 27 proteins and 2,966 residues—450 (15.2%) of the EFR class and 2,516 (84.8%) of the LFR class. Due to the nature of the HDX experiments no data can be obtained for proline residues which feature no amide group susceptible to HDX [39], rendering them LFR in any case. Annotation of functional residues was performed using the SIFTS [96] and UniProt [97] resource. We collected entries in the “Function” and “Family & Domains” section when they were associated to protein function. For 22 proteins an annotation of functional residues existed, totaling in 2,490 residues—152 (6.1%) classified as functional and 2,338 (93.9%) as non-functional. Information used from the Start2Fold database can be found in S3 File. Residues annotated as functional are summarized in S4 File which contains a description of the matched XML tags of functional residues.

### Statistical analysis of the Start2Fold dataset

EFR and functional residues are almost entirely disjoint in the specific case of both aaRS superfamilies. We statistically analyzed the more general Start2Fold database [41] to assess whether this separation is a common theme in protein structures.

Various features were used to describe residues of the dataset and it was tested whether these features differ significantly between EFR and LFR as well as EFR and functional residues. In both cases, *p*-values were computed on the subset of buried residues (RASA less than 0.16 [60]), because EFR tend to be buried in the hydrophobic core of proteins [23] whereas functional residues are likely exposed to the solvent. All tables present the average and standard deviation of the considered features for all residues and the corresponding *p*-value for the subset of buried residues. Also, the *p*-value for buried residues is used when the level of significance is stated. Dependence of distributions of real-valued variables was tested by the Mann-Whitney U test. Dependence of distributions of count variables was tested using the Dunn test with Bonferroni correction. Throughout the manuscript, * corresponds to significant *p*-values <0.05 for the Mann-Whitney U and *p*-values <0.025 for the Dunn test. A variation of conventional boxplots is used, which depict a notch around the median of the distribution. This notch corresponds to the 95% confidence interval and allows to visually assess whether the medians of two boxes are similar. No overlap of notches indicates that both medians differ substantially [98].

### Graph representation and analysis

Protein structures are commonly represented as residue graphs: amino acids constitute the nodes and contacts between residues are represented as edges [61, 78, 99]. This allows a scale-invariant characterization of the neighborhood relation of individual amino acids in the context of the whole protein [99].

In this study, amino acids constitute the nodes of a graph, whereas covalent bonds and residue contacts are represented as edges. Residues were considered in contact when their C_*α*_ atoms were less than 8 Å apart. Furthermore, contacts were labeled as either local (i.e. the separation in sequence is less than six) or tertiary (i.e. sequence separation greater than five) [26]. This distinguishes contacts stabilizing secondary structure elements and those which represent contacts between secondary structure elements. The set of distinct neighborhoods of a node is defined as all adjacent nodes which do not share any local edge to any element of the set. Betweenness is defined the number of shortest paths on the graph passing through a specific node, normalized by the number of node pairs [61, 100]. Closeness of a node is defined as the inverse of the average path length to any other node [81]. The clustering coefficient of a node is the number of edges between its *n*_*k*_ adjacent nodes divided by the maximal number of edges between *n*_*k*_ nodes: 0.5 ⋅ *n*_*k*_ ⋅ (*n*_*k*_ − 1) [61].

### Energy Profiling

Energy Profiles were calculated from structure and predicted from sequence according to the methodology used in the eQuant web server [29, 30]. Energy Profiles represent a protein’s complex three-dimensional structure as one-dimensional vector of computed energies. Thereby, the surroundings of each residue are characterized by one energy value. Therefore, the frequencies of an amino acid to occur buried or exposed to the solvent were determined. Using the inverse Boltzmann law, the fraction of both states can be expressed as pseudo-energy. The energy of a residue can then be computed by summing up the corresponding pseudo-energies of all interacting residues. Residues were considered in contact, when the distance of their *C*_*β*_ atoms was less than 8 Å [29]. Low computed energies occur for hydrophobic amino acids which are stabilized by many contacts. Thus, this approach is a valuable feature to assess the stability of individual residues as well as their interactions with their spatial neighborhood.

### Feature computation

RASA values were computed by the algorithm of Shrake and Rupley [101]. Buried residues are defined as those with RASA values less than 0.16 [60]. Non-covalent residue-residue contacts were detected by PLIP [59]. Secondary structure elements were annotated using DSSP [102]. For both ASA and secondary structure element annotation the BioJava [103, 104] implementations were used. The loop fraction is defined as fraction of unordered secondary structure in a window of nine residues around the evaluated amino acid [105]. This yields a fraction, where high values are tied to regions of high disorder, whereas amino acids embedded in *α*-helices or *β*-sheets result in scores close to zero. The centroid distance of a residue is the spatial distance of its centroid to that of all atoms. The terminus distance is the minimal number of positions to traverse to reach N- or C-terminus divided by the total number of residues. Evolutionary information as well as evolutionary co-variation scores for the Start2Fold dataset were computed using the EVfold web server [86, 87]. The evolutionary information is based on the MSA of homologues automatically retrieved for the query sequence and expresses how conserved a column in this MSA is.

Data integration was performed by a Java library publicly available at https://github.com/JonStargaryen/jstructure.

## Supporting information

S1 Fig Correlation matrix of computed features. Depicts correlations of analyzed correlation. The bigger the circle, the higher the association of both variables. Blue refers to positive correlation, whereas red represents a negative correlation.
(TIF)

S2 Fig Network descriptors. Depiction of the used network descriptors: betweenness, closeness, clustering coefficient, and distinct neighborhood count.
(TIF)

S1 Table Summary of the aaRS dataset. Sequence conservation [93, 94] and EFoldMine [9] predictions for the aaRS protozyme regions [46–48] are presented. Encompassed are the average values for all residues, residues in the protozyme region, for positions predicted to be EFR, functional residues, ATP binding residues, and amino acid binding sites.
(XLSX)

S2 Table Statistical characterization of EFR. For each presented feature the mean (*μ*) and standard deviation (*σ*) of both the EFR and LFR category is reported. It was tested whether the differences of a feature between EFR and LFR state is significant. *p*_buried_ refers to the *p*-value of the test on residues buried according their RASA value, this was done because EFR have a tendency to be located in the core of a protein and without filtering all differences are significant. The Mann-Whitney U test was used for real-valued variables, whereas the Dunn test was used for count variables (indicated by #). 2,966 residues in 27 proteins from the Start2Fold database [41] were analyzed.
(XLSX)

S3 Table EFR dataset summary. Summarizes identifiers [23] of each entry as well as the number of residues in the corresponding protein chain, the number of EFR and functional residues as well as the cardinality of the intersection of both sets. To assess the relevance of the observed intersection it was compared to the expected intersection. Negative shift values occur when the observed intersection is smaller than that expected by the individual frequencies of EFR and functional residues. Positive values are observed when the overlap is more pronounced than to be expected. Proteins not containing any functional residues according to UniProt [97] are marked with dashes.
(XLSX)

S4 Table Comparison of EFR and functional residues. For each presented feature the distribution of values is compared between functional and non-functional residues as well as EFR and functional residues. The corresponding *p*-values and significance level are stated for buried residues. Mean values are shown for EFR (*μ*_early_) and functional residues (*μ*_func_). The Mann-Whitney U test was used for real-valued variables, whereas the Dunn test was used for count variables (indicated by #). 2,490 residues in 22 proteins from the Start2Fold database [41] were analyzed.
(XLSX)

S1 File Detailed description of aaRS class I structures. For each renumbered position, it is stated whether it is functional [48] or an EFR. Furthermore given are the sequence conservation [93, 94], the number of backing sequences [48], and the average EFoldMine score [9].
(CSV)

S2 File Detailed description of aaRS class II structures. For each renumbered position, it is stated whether it is functional [48] or an EFR. Furthermore given are the sequence conservation [93, 94], the number of backing sequences [48], and the average EFoldMine score [9].
(CSV)

S3 File Start2Fold dataset as JSON file. Machine-readable JSON version of the dataset. Provides protein name, Start2Fold identifier, PDB identifier, UniProt identifier, number of EFR, range of residues numbers, and the secondary structure element composition for each dataset entry.
(JSON)

S4 File Start2Fold dataset as table. Summary table of all protein chains used for the analysis. Provides Start2Fold identifier, PDB identifier, evaluated HDX experiments, number of EFR, UniProt identifier, and identifiers of functional residues derived from UniProt. The last column contains the features in the UniProt XML file considered functional for this entry.
(LIST)

S5 File Table of computed features for the Start2Fold dataset. Contains for all residues the set of computed features as well as the annotation of Early Folding and functional residues.
(CSV)
