# Supplementary figures and images for: Characterizing the relation of functional and Early Folding Residues in protein structures using the example of aminoacyl-tRNA synthetases
Source: PLoS One. 2018 Oct 30;13(10):e0206369. doi: 10.1371/journal.pone.0206369 (PMC6207335; doi:10.1371/journal.pone.0206369)

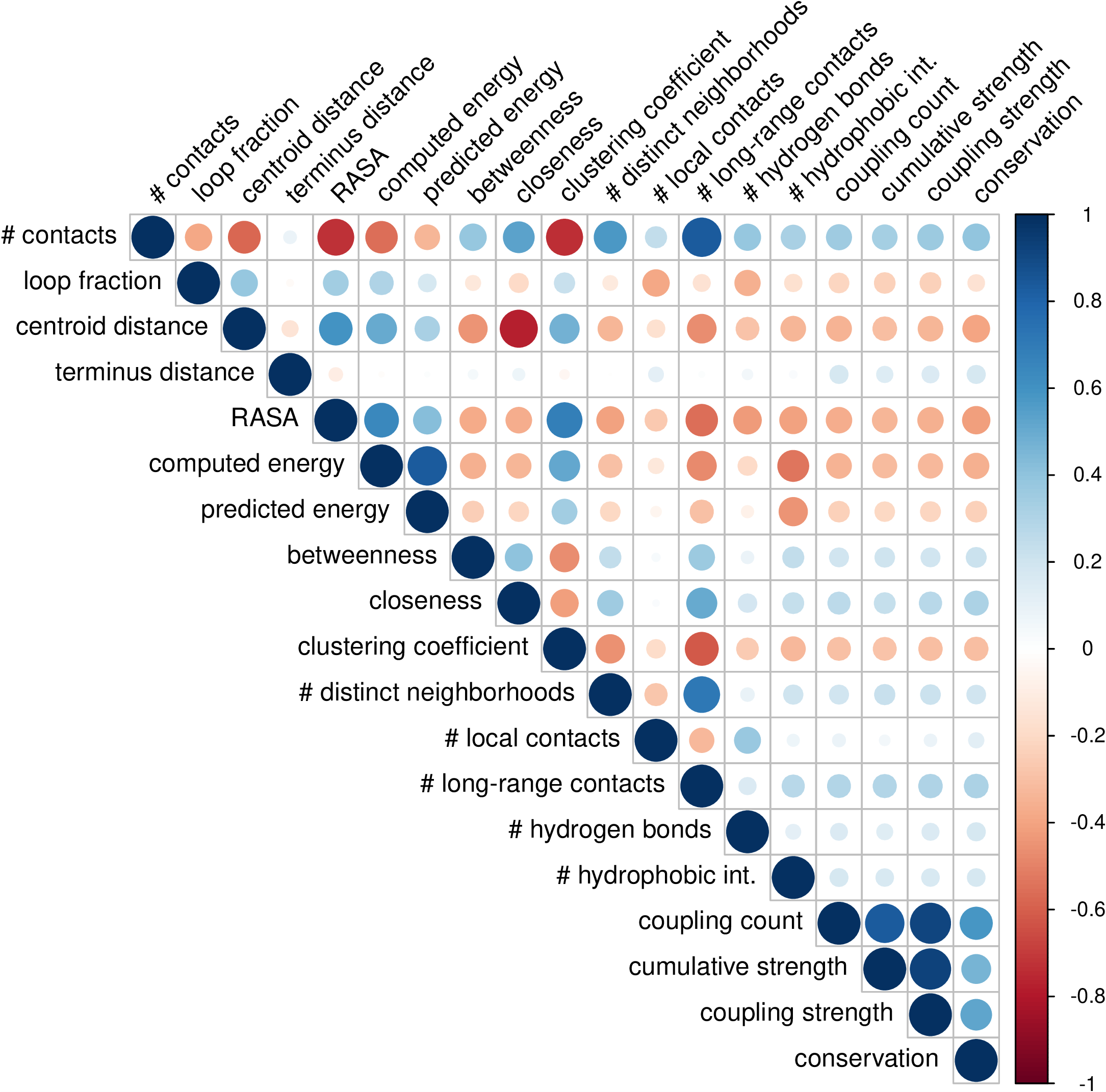

Supplement: S1 Fig — Depicts correlations of analyzed correlation. The bigger the circle, the higher the association of both variables. Blue refers to positive correlation, whereas red represents a negative correlation. (TIF) [file pone.0206369.s001.tif]

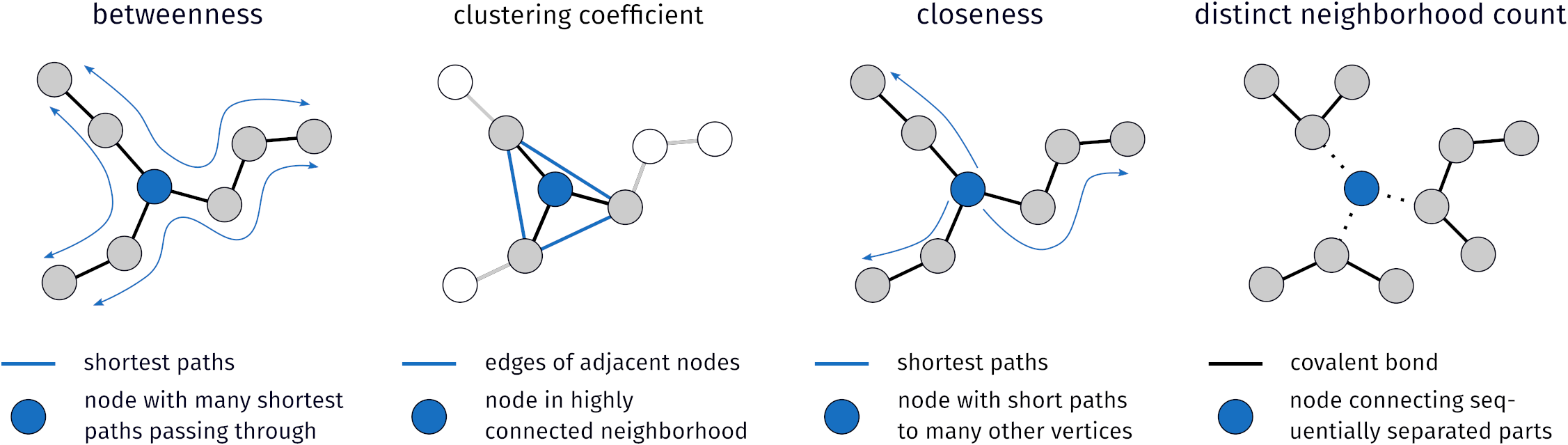

Supplement: S2 Fig — Depiction of the used network descriptors: betweenness, closeness, clustering coefficient, and distinct neighborhood count. (TIF) [file pone.0206369.s002.tif]
